# A Social Reform from the Kitchen

**Published:** 1890-02

**Authors:** Edward Atkinson


### A SOCIAL REFORM FROM THE KITCHEN.

It is a well-ascertained fact that, with respect to about ninety per cent. of the community, the price paid for food comes to one-half the income or more. After this food is bought how much of it is wasted in bad cooking? How much human force is wasted in consequence of bad cooking? How much does dyspepsia or indigestion, caused by bad cooking, impair the working capacity of the people of the United States and diminish their product? Can five cents' worth per day be saved? Is not that a very insufficient measure of the difference between a poor, wasteful cook and a good, economical one? If five cents a day can be saved on food and fuel, while at the same time that which is bought and cooked may be converted into more nutritious and appetizing food, the difference in each community of 6,000 people would be $109,500 a year, or about nine per cent. of the total product of the typical community, which we have assumed to be $1,200,000 a year in gross. When the attention of the labor reformer is brought down from grand schemes for altering the whole constitution of society, by act of Congress or of the State Legislature, to the simple question of how each person, each family, or each community may better itself under existing conditions, great progress will have been made in solving all the problems which are now pending.—Edward Atkinson in the April *Forum*.

